# Lived experiences of obstetric anal sphincter injury among women from ethnically diverse backgrounds in the UK: a qualitative study

**DOI:** 10.3389/fgwh.2026.1966289

**Published:** 2026-10-07

**Authors:** Olufisayo Olakotan, Jennifer N. W. Lim, Mina Bhavsar, Farah Siddiqui, Rabina Ayaz, Gillian O'Brady Henry, Thillagavathie Pillay

**Affiliations:** 1 Department of Neonatology, University Hospitals Leicester NHS Trust, Leicester, United Kingdom; 2 Faculty of Education, Health and Wellbeing, University of Wolverhampton, Wolverhampton, United Kingdom; 3 Leicestershire and Rutland Local Maternity and Neonatal Systems, Leicester, United Kingdom; 4 Department of Obstetrics, University Hospitals Leicester NHS Trust, Leicester, United Kingdom; 5 Leicestershire Partnership NHS Trust, Leicester, United Kingdom; 6 Faculty of Science and Engineering, University of Wolverhampton, Wolverhampton, United Kingdom; 7 University of Leicester College of Health Sciences, Leicester, United Kingdom

**Keywords:** health care, lived experiences, maternal care, obstetric anal sphincter injury (OASI), perinatal health inequalities

## Abstract

**Background:**

Obstetric anal sphincter injury (OASI) is a severe childbirth-related injury that can have significant physical, psychological and social consequences, with potentially lasting effects on women's quality of life. Understanding the lived experiences of ethnic minority women affected by OASI is important for improving the care and support provided to them.

**Aim:**

This study explores the lived experiences of ethnic minority women with OASI in Leicester, UK, with a focus on their childbirth experiences, healthcare challenges, and the broader impact on their overall well-being.

**Methodology:**

A focus group discussion (FGD) was held virtually with underrepresented women from Leicester who had experienced OASI in the past one to five years. The discussion was recorded and transcribed. Invitations to participate in the study were sent out via email through the local maternity and neonatal voices partnership in Leicester, UK. The data were analysed using the six-step thematic analysis approach by Braun and Clarke.

**Results:**

Ten women from underrepresented groups identified several key themes such as childbirth experiences, complications and reflection, societal influence and childbirth choices, cultural silence, stigmatization, and mental health issues. The women reported feeling uninformed and unprepared about possible complications and risks such as third and fourth-degree tears, resulting in traumatic birthing experiences, aggravated by inadequate healthcare communication and support from healthcare providers.

**Conclusion:**

There is the need for future research to integrate physical, physiological and mental health support, as well as sustained follow-up into OASIS care bundle. This will essentially support women's health and well-being during the perinatal period.

## 1 Introduction

Obstetric anal sphincter injury (OASI) is a severe form of childbirth-related perineal trauma involving partial or complete disruption of the anal sphincter complex and is classified as third- or fourth-degree perineal tears (1, 2). Third-degree tears are further classified according to the extent of sphincter involvement: a 3a tear involves less than 50% of the external anal sphincter (EAS) thickness; a 3b tear involves more than 50% of the EAS thickness; and a 3c tear involves both the EAS and internal anal sphincter (IAS). A fourth-degree tear involves disruption of the EAS and IAS and extends through the anorectal mucosa (3). Contemporary United Kingdom (UK) data indicate a median OASI rate of approximately 3.1%, with rates substantially higher among primiparous than multiparous women (6.4% versus 1.2%, respectively) (4). In a recent UK tertiary maternity-unit cohort, 3a tears accounted for 53.0% of OASI cases, followed by 3b tears (35.2%), 3c tears (8.9%) and fourth-degree tears (3.0%) (5). Although reported rates of OASI increased considerably in England between 2000 and 2012, improved recognition and the introduction of standardised classification have been identified as important contributors to this historical increase in reported incidence (6).

The occurrence of OASI is influenced by several maternal, fetal and intrapartum factors. Evidence from the UK evidence has identified primiparity, instrumental vaginal birth, Asian ethnicity, higher birthweight and shoulder dystocia as important risk factors (5). OASI can result in significant short- and long-term physical morbidity (7–9). For example, women reported experiencing panic disorders, postpartum depression, difficulty discussing intimate issues with family and friends, thoughts of suicide, and symptoms related to post-traumatic stress disorder following OASI (1, 7). Some women linked their mental distress to the complicated perineal laceration during childbirth and the challenges of postpartum recovery (7). Many women reported a lack of sufficient information about OASI before and after childbirth, pain expectations, healing process, possible complications, post-OASI follow up with healthcare professionals and whom to contact in case of complications (7–9). On the other hand, women who engaged with healthcare services reported that their problems and pains were neglected by the caregivers and OASI advice were perceived unhelpful (1, 7). They appreciate meeting health care staff that listens to them and takes their questions and worries seriously (8).

Also, the attitude of healthcare professionals during childbirth impacted how women experienced and understood OASI (9). At times, these interactions were reported as insensitive and inappropriate (9). For instance, during the suturing process, women recalled the facial expressions of the healthcare providers, as well as how midwives and obstetricians spoke around and about them, which made the women feel vulnerable and exposed during the procedure (9). While OASI disproportionately affects underrepresented groups, exacerbated by disparities in access to care and support, many women reported difficulty navigating treatment pathways, and contacting the right healthcare professionals for routine check-ups and follow-ups related to perineal tears (7–9).

Despite growing recognition of women's experiences following OASI, less is understood about how these experiences may be shaped by cultural and social contexts (10). Cultural beliefs, social expectations, understandings of psychological distress, and attitudes towards help-seeking may influence how women interpret their experiences, communicate their needs, and engage with maternity and perinatal services (11). For women from culturally and ethnically diverse backgrounds, these factors may intersect with wider inequalities in access to appropriate information, communication, and healthcare support, including language barriers, difficulties navigating maternity services, and experiences of culturally insensitive or discriminatory care (11) (12), (13),. Understanding these perspectives is therefore important for developing maternity and postnatal services that are responsive to women's individual, cultural, and psychosocial needs (13).

Leicester, a highly diverse city in the UK where approximately 50% of mothers giving birth in hospitals are from non-British Caucasian backgrounds, provides an important setting for this exploration (14). Understanding the specific challenges faced by women from culturally diverse backgrounds in Leicester is crucial to improving healthcare support, addressing disparities, and ensuring that postnatal care is sensitive to the needs of all women (15, 16). This study explores the lived experiences of women with OASI from diverse cultural backgrounds in Leicester, UK, focusing on their childbirth experiences, healthcare challenges, and the broader impact on their well-being.

## 2 Methods

### 2.1 Setting

The focus group discussion (FGD) was organized jointly by the Local Maternity and Neonatal System (LMNS) at the Leicester Royal Infirmary (LRI), which is part of the University Hospital of Leicester (UHL) partnered with Leicester Mammas (17), a Leicester-based organization that supports mothers and babies with breastfeeding and other needs. LRI, a level 3 care facility, handles approximately 10,000 births annually and serves as the base for the children's hospital, which provides treatment for various childbirth-related conditions.

### 2.2 Participants and recruitment

A community organisation known as Leicester Mammas, which supports pregnant women, new mothers and their families, emailed the study invitation and accompanying flyer to sixteen women from underrepresented groups within its network (17). The invitation specifically sought women who had experienced a third- or fourth-degree perineal tear within the previous one to five years and provided information about the purpose of the study, the nature of participation, and participants’ right to withdraw at any time. During the FGD, participants self-confirmed that they had experienced a third- or fourth-degree perineal tear and that the affected childbirth had occurred within the previous one to five years. Eligibility relating to the tear and its timing was therefore based on participants’ self-report and was not independently verified through medical records.

#### 2.2.1 Eligibility criteria

The inclusion criteria were: women aged 18 years or older; membership of an underrepresented group targeted by the study; experience of a third- or fourth-degree perineal tear within the previous one to five years; sufficient English proficiency to understand the study information and participate in the FGD; and willingness and ability to provide informed consent. Women were excluded if they were younger than 18 years, had no qualifying history of a third- or fourth-degree perineal tear within the specified period, were unable to participate sufficiently in English, or were unable or unwilling to provide informed consent.

#### 2.2.2 Confidentiality and data management

To maintain confidentiality, participants were assigned unique study codes, which were used during transcription, data analysis and reporting. Personally identifiable information, including participants’ names and specific locations, was removed from the transcripts and was not included in the manuscript. Consent forms containing identifiable information were stored separately from the anonymised research data. Audio recordings and transcripts were stored securely on the university drive, with access restricted to authorised members of the research team. Anonymised demographic information and participant quotations were reported only with participants’ consent, and care was taken to ensure that individuals could not be identified from the information presented in any resulting publications.

### 2.3 Data collection

On June 9, 2023, a focus group discussion (FGD) was conducted to explore the experiences and perspectives of women who had experienced severe perineal tears**.** Written informed consent was obtained from all participants before the FGD. Written informed consent was obtained from all participants before the FGD. Demographic information, including age, ethnicity, languages spoken, number of children and history of third-degree perineal tear, was collected from participants before the FGD using a brief structured demographic questionnaire.

A semi-structured topic guide, developed by obstetricians within the research team and aligned with the study objectives, was used to guide the FGD. The guide covered participants’ childbirth experiences, experiences of severe perineal tears, and the wider physical, psychological, cultural and social impact of their experiences. The semi-structured format provided consistency across the areas explored while allowing participants to elaborate on issues that they considered important and enabling the facilitator to use follow-up prompts where appropriate.

The FGD was preceded by an educational session led by FS, a neonatal consultant, which provided background information on disparities in third- and fourth-degree tears among ethnic minority women. This was followed by presentation of data from UHL's perinatal health inequality dashboard. The rationale for providing this information before the FGD was to establish a common factual context for the subsequent discussion, recognising that participants might have different levels of prior knowledge about OASI and reported ethnic disparities (18). This approach was informed by principles of deliberative discussion focus groups, in which relevant information may be provided before discussion of complex health topics to support informed participation, and by established focus-group methodology that permits the use of stimulus material to focus and facilitate discussion (19). The educational session was not itself a source of qualitative data; however, its potential to influence or frame the subsequent discussion was recognised and is acknowledged as a study limitation.

Following the educational session, TP, also a neonatal consultant, facilitated the two-hour FGD virtually via Microsoft Teams. The discussion was audio recorded with participants’ consent and subsequently transcribed for qualitative analysis. The FGD was conducted in English and interpreter support was not used. Given the sensitive nature of the topic, participants were reminded that they could decline to answer questions, pause their participation or withdraw at any time if they experienced discomfort or emotional distress. A psychologist was not present during the FGD, and no formal distress pathway was established for the study. Participants were given an opportunity to raise additional concerns at the end of the session and were signposted to appropriate healthcare services where required.

### 2.4 Data analysis

The FGD transcript was analysed using Braun and Clarke's six-phase thematic analysis approach (20). The analytic process was iterative, with movement between phases as the analysis developed. During the first phase, OO and TP read and re-read the complete FGD transcript to become familiar with the participants’ accounts and noted initial observations relevant to the study objectives. Sections of the transcript that captured meaningful aspects of participants’ experiences were systematically coded. NVivo was used to organise and manage the transcript, codes and associated data extracts. Related codes were compared and grouped according to shared meanings and patterns, from which candidate themes were developed. Related codes were subsequently grouped and developed into themes.

Afterwards, OO and TP reviewed the candidate themes against the coded extracts and the complete FGD transcript to assess whether they adequately represented participants’ accounts and addressed the study objectives. Themes were revised, combined or separated where necessary. The scope and central meaning of each theme were further refined, and clear theme names were developed to reflect the experiences represented within the data. In the final phase, themes were organised into a coherent analytic narrative and illustrated using relevant participant quotations. The wider research team discussed the developing analysis throughout the process, with different interpretations considered through discussion to strengthen the depth and coherence of the analysis. The final themes were reviewed in relation to the study objectives and the overall accounts provided by participants.

### 2.5 Reflexivity

The research team comprised members with clinical, academic and maternity-service backgrounds. Several members had professional experience within maternity and neonatal healthcare, including FS and TP, who were neonatal consultants. TP facilitated the FGD, while OO and TP were involved in data collection, analysis and interpretation. The team recognised that their clinical backgrounds, professional knowledge of maternity care and prior awareness of inequalities in OASI could influence the focus of enquiry, interactions with participants and interpretation of the data. Particular consideration was given to the possibility that participants might respond differently when discussing healthcare experiences with a clinician. During analysis, developing themes were repeatedly reviewed against the original FGD transcript and discussed within the wider research team to ensure that interpretations remained grounded in participants’ accounts rather than being driven primarily by clinical assumptions. This supported a transparent and critically reflective interpretation of the data while recognising the influence of researcher perspectives in qualitative research.

## 3 Results

Ten women from diverse ethnic backgrounds responded and participated in the FGD following invitations sent to sixteen women. Among participants who reported their age, ages ranged from 25 to 40 years. Their demographic characteristics, including age, ethnicity, languages spoken, number of children and experience of third-degree perineal tears, are presented in Table 1. The analysis identified several themes, including childbirth experiences, complications and reflection, societal influence and childbirth choices, cultural silence and stigmatisation, mental health barriers, insufficient prenatal education, childbirth trauma and postpartum support.

**Table 1:** Demographics of participants.

Focus group session						
Participants	Age	Experienced of third-degree tears	Number of such experiences	Number of children	Ethnicity	Languages spoken
KC	40	Yes	One	Two	Indian	English, Gujarati,
HB	33	Yes	One	One	Pakistani	English, Urdu
AD	30	Yes	One	One	Asian	Gujarati, English, Punjabi, Hindi
RP	36	Yes	One	Two	White	Bulgarian, English
NT	28	Yes	One	Two	Indian	English
BP	40	Yes	One	Three	Indian	English, Gujarati
HV	25	Yes	One	One	Indian	English, Gujarati
YH	Nil	Yes	One	One	Indian	Indian, English, Gujarati
NO	30	Yes	One	One	African	English
SS	33	Yes	One	Two	Indian	English, Urdu

### 3.1 Childbirth experiences and medical interventions

Participants’ accounts of childbirth reflected experiences of rapid clinical decision-making, medical intervention and a perceived lack of time to physically and emotionally prepare for birth. Across these narratives, women described labour as progressing quickly and, in some cases, unexpectedly, contributing to feelings of reduced control over the birth process. One participant reflected on the speed of her labour following induction and epidural administration: “*I have my own experience because my own labour wasn't as long. I felt like if there had been enough time to get my body ready it would have been a bit more different. So it was that induction that I had, I had an epidural as well. I think within that time, it was probably about 10–15 min or less..the next thing I was told to start pushing” (HV).* For others, the experience was compounded by serious complications affecting both mother and baby: “*There has to be an emergency for me because the baby was not crying after birth, so the baby has to be resuscitated. I was losing quite a lot of blood; the examiner said that I have quite an extensive third-degree tear” (HB).*

A further pattern across participants’ accounts was retrospective questioning of the circumstances surrounding childbirth. Women attempted to make sense of why the tear had occurred and whether aspects of labour management or their own actions might have contributed to the outcome. This reflection was sometimes accompanied by uncertainty and self-blame. One participant questioned whether being instructed to push immediately after reaching full dilation may have affected her outcome: “*I've been wondering, is it because I pushed immediately even though I was 10 cm dilated? Now that I think about it, if I was given more time to kind of prepare instead of just pushing out, I don't know, maybe the outcome would have been different"(KC).* Another described continuing to question possible maternal factors and expressed concern about future childbirth: “*I keep thinking to myself, what is going to happen again when I'm about to have another baby. You just start questioning yourself and wonder what maternal factors could have contributed to this number of tears"*(SS).

### 3.2 Societal influence around childbirth choices

Participants described childbirth choices as being shaped not only by their own preferences but also by wider social expectations about how women should experience and manage labour. Epidural use was one area in which women described feeling influenced by perceptions of what was considered acceptable or appropriate. One participant recalled initially viewing epidural use negatively because of these expectations*: “I thought it was beneath me to have an epidural..* *that's the only thing I'm going to take because that is deemed as appropriate”* (AD).

Across the accounts, however, experience of subsequent childbirth appeared to strengthen some women's confidence in prioritising their own needs over external expectations. Greater familiarity with childbirth enabled some participants to exercise more control over decisions about pain relief. As one woman explained: “*Third time round I had control and asked can I please have an epidural..* *and then it was amazing” (NO).*

For some participants, this shift in perspective extended beyond their own experiences and shaped the advice they would give to other women. They emphasised individual choice and encouraged women to make decisions based on their own comfort rather than societal judgement: “*Don't, please don't hesitate to take an epidural and nobody needs to know if you think that..* *you do whatever you like and you don't even need to tell anybody about that” (BP)*

### 3.3 Cultural stigma around maternal and mental health

Across participants’ narratives, cultural expectations influenced how openly women felt able to discuss childbirth experiences, emotional difficulties and mental health. A recurring pattern was the expectation that difficult or distressing experiences should be managed privately rather than openly discussed. One participant described this expectation within her family: “*With the family I come from, you hear things like, ‘Don't talk about it.’ It's not something that you should be talking about, just deal with it” (NT).*

Participants from Asian backgrounds particularly described cultural expectations of silence and endurance when dealing with personal difficulties. As one participant explained: “*I think in our culture and the Asian background, nobody just talks about anything that they go through because they just have to get on with it”* (YH).

The expectation to remain silent was also evident in participants’ accounts of mental health. Women described stigma as making emotional and psychological difficulties particularly difficult to acknowledge or discuss. *One participant stated: “Mental health issues cannot be discussed, especially when it related to health and care” (SS).* Importantly, these experiences were not confined to one cultural background. Similar expectations were described by a participant from an Eastern European background, suggesting that cultural norms surrounding resilience and silence were experienced across different communities: “*Our culture is very similar in the sense that there is no such thing as mental health and that you are a woman, you should just get on with it; the stigma is there” (RP)*

### 3.4 Gaps in prenatal education

A consistent pattern across participants’ accounts was a perceived lack of antenatal information about severe perineal tears. Women described entering childbirth without sufficient understanding of the possibility of third- or fourth-degree tears, their severity or the potential consequences. This lack of information contributed to feelings of being unprepared for what occurred during and following childbirth. One participant explained: “*From my point of view and experience, no one ever spoke to me about those potential tears or the severity and the different types. I was only told about the controlled cut that they might do. That was all I was prepared for” (BP).* Another similarly described a wider lack of preparation for both severe tears and the postpartum period: “*No, we don't get told that we're going to have third degree tears. whatever happens in the labour room happens in the labour room; also, there's never anything on the fourth trimester..* *what I'm hearing is that we were unaware of what was about to happen” (NO).*

However, participants did not necessarily suggest that women should receive unrestricted information about every possible complication. Rather, their accounts reflected a need for balanced and appropriately communicated information that could improve preparedness without generating unnecessary fear. This tension between being sufficiently informed and becoming overwhelmed was captured by one participant: “*So, this is like a balance that needs to be struck. Because you can't tell someone everything, making them go into labour scared. Just inform them about possible outcomes so they can be a bit more mentally prepared for it” (KC).*

### 3.5 Enduring psychological impact of traumatic childbirth

Participants described the psychological consequences of traumatic childbirth as extending well beyond the immediate birth experience. Their accounts reflected persistent emotional distress that affected everyday life, relationships and subsequent encounters with healthcare. For some women, the experience was understood in terms of continuing trauma. One participant described the impact on both herself and her partner: “*I can say that I just suffered PTSD for probably a good few months, it affected my husband and other daily activities” (RP).*

The circumstances surrounding childbirth also shaped how participants made sense of their trauma. For some, restrictions and disruptions associated with the COVID-19 pandemic intensified feelings of distress and loss during childbirth. One participant explained: “*I was rushed so I didn't really have skin to skin contact, it was during covid as well… I found very traumatic… but this time I was just very determined that I know what happened and was the worst-case scenario” (YH).*

Participants’ accounts also suggested that traumatic childbirth could influence subsequent interactions with healthcare. For some women, memories of the birth remained sufficiently powerful to affect their ability to tolerate examinations or follow-up care. One participant reflected*: “Because I was at home for so long and maybe it was a traumatic experience for my body to experience contractions for that long. I was traumatized… It took me about three hours for me to lay on the bed when I for follow up and I couldn't let her do it (NT).”*

The psychological consequences could remain unresolved for several years, with some participants describing a prolonged process of recognising and seeking help for their experiences. As one participant stated: “*So, it's a very long five years trying to get to the bottom of this… I realized something's not right, and I've been through trauma, and I definitely need some sort of counselling”(SS).*

### 3.6 Poor communication and lack of support

A recurring pattern across participants’ narratives was feeling insufficiently informed, listened to or supported by healthcare professionals during childbirth and recovery. Women described instances in which their concerns appeared to be underestimated or their need for support was not adequately recognised, contributing to feelings of vulnerability during an already distressing experience. One participant recalled: “*I was taken in and was told that I wasn't in labour at that point, but actually was near full dilation. The midwife that was supposed to support me left me on the side of the room..* *she just stood on the other side of the room and watched"(KC).*

Communication difficulties were also reflected in the language used by healthcare professionals. Participants described struggling to understand clinical terminology during childbirth, particularly when experiencing pain, exhaustion or distress. This reduced their ability to fully understand what was happening to them at the time. One participant explained: “*You don't really grasp the terminology they're using at the time; mentioning words like third-degree tear, catheter, because you're kind of out of it as well. I was left trying to decipher what was happening and why"(AD).*

### 3.7 Navigating conflicting advice in infant feeding

Participants described receiving inconsistent advice about infant feeding from different healthcare professionals during the postnatal period. Rather than providing reassurance, conflicting recommendations contributed to uncertainty at a time when women were simultaneously recovering from childbirth and managing the demands of caring for a newborn. One participant described receiving different advice from midwives and health visitors: “*Midwives will be like don't give him formula yet, and then health visitors were like give him formula… so it was like two different words, and then you are like who do we follow” (HB).*

The complexity of decision-making was further increased when advice from family members differed from professional recommendations. Although family advice was generally intended to provide support, participants described having to negotiate multiple and sometimes contradictory sources of guidance. As one participant explained: “*Family obviously will do what is best for you by simply giving you advice that contradicts the midwives and health visitors” (BP).*

## 4 Discussion

Our study explored the lived experiences of women with third- and fourth-degree tears (OASI) in Leicester, UK, highlighting childbirth experiences, healthcare challenges, and the broader impact on their overall well-being. Many women experienced lasting psychological trauma, including anxiety, PTSD-like symptoms, and self-blame. Gaps in healthcare communication and support were also evident, with reports of dismissive attitudes, unclear medical explanations, rushed interventions, and conflicting advice on infant feeding. Additionally, cultural stigma discouraged discussions on maternal mental health and childbirth complications.

The participant's description of a rapid labour, induced and assisted with an epidural, echoes existing concerns in obstetric practice regarding the potential risks of accelerated labour. The issues raised in studies underscore a growing body of evidence suggesting that while interventions are sometimes medically necessary and beneficial, they can also disrupt the natural progression of labour (21, 22). Furthermore, participants’ reflections suggesting self-blame or a sense of responsibility for the complications are a common psychological response found in mothers who have undergone traumatic childbirth experiences. This highlights the need for support that addresses not only physical recovery but also mental and emotional well-being (23).

The psychological aftermath of traumatic childbirth, including post-traumatic stress disorder (PTSD) and its impact on both the mother and her partner, has long-lasting implications for women's health and well-being (24, 25). The intensity of the trauma can be linked to factors such as the duration of labour and physical strain, echoing findings from other research indicating that prolonged or complicated labour can increase the risk of psychological trauma (26, 27). The long-lasting nature of this trauma, requiring years to address, aligns with studies emphasizing the need for long-term mental health support for affected women experiencing traumatic childbirth (28–30). This aligns with recommendations from previous studies advocating for the integration of mental health screening and support into postpartum care (30, 31). Moving forward, healthcare systems should prioritize enhancing awareness, early detection, and intervention for childbirth-related trauma, ensuring that both physical and psychological needs are addressed in the postpartum period.

In some cultures, the reluctance to openly discuss personal challenges, including childbirth and mental health, stems from deep-rooted beliefs that prioritize endurance and discretion over open dialogue about difficulties (32). This hinders women from seeking necessary support, especially for mental health issues, which are often stigmatized. To address these challenges, healthcare providers need to adopt culturally sensitive practices (33, 34). This involves understanding and respecting cultural differences in health-seeking behaviours and communication styles. For instance, a mixed-methods study found that women preferred a discursive (and therefore inclusive) communication style during the procedure and described the importance of compassion and communication (35). Additionally, integrating cultural competence into healthcare is crucial for providing effective and inclusive care, and the role of cultural factors in health-seeking behaviours is significant (36, 37). By acknowledging and accommodating these cultural nuances, healthcare systems can better support diverse populations, particularly in managing sensitive issues such as mental health and childbirth experiences.

The study findings raise the critical question of whether women should receive information about potential risks and complications like third-degree perineal tears during pregnancy or not. Studies evaluating the effectiveness of preparation classes have shown that information about the postnatal period is often lacking, despite it being a recognized goal of antenatal care (28, 38). Providing women with overwhelming information may induce fear, anxiety, leading to difficulty in decision making and confusion (28). On the other hand, lack of information about pregnancy associated risks may be distressing for the women when faced with complications (39, 40). A balance approach means that sensitivity and personalization should be taken into consideration when communication risks to women. This means that the information should be tailored to meet the needs of individuals and also provide opportunities for women to discuss their concerns with healthcare professionals (39, 40).

Situations where midwives advocate for exclusive breastfeeding while health visitors may suggest introducing formula create a challenging dilemma for parents. A similar study highlighted the challenges of breastfeeding, noting that difficulties often arose due to inadequate or conflicting advice from health professionals (41). Another study reported that midwives directly contradicted and discredited the advice from other midwives, with some participants being told to not listen to midwives (42). Examples of recurring subject areas where inconsistent guidance was observed include advice on positioning, the use of nipple shields, the practicalities of feeding on demand, and the interpretation of infant sleep and settling issues in relation to breastfeeding advice (42). For some women, the issue was being presented with an overwhelming number of solutions to a problem, frequently altering strategies without allowing sufficient time to evaluate their effectiveness, and not maintaining a consistent approach for more than one feeding session (42, 43). To overcome this situation, women talked about doing their own research to address the conflicting advice received from midwives, family, and friends regarding whether or not to continue breastfeeding (44). Hence, women need detailed guidance on breastfeeding, including expectations, practical assistance with positioning, valuable advice, recognition of their feelings and experiences, and supportive encouragement (42, 43).

The literature often mentions the OASI care bundle as part of evidence-based practices focusing on maternal aspects, primarily aiming to prevent physical injuries during childbirth (6, 45). Moreover, existing literature and subtle feedback from women in our study highlight the limitations of the OASIS care bundle in addressing vital aspects of women's health, such as psychological support, long-term follow-up, and mental health care following third and fourth degree tears (45–47). This critique aligns with research indicating that supportive components should be integrated into perinatal health services to meet women's needs, highlighting a gap between current practices and patient needs (48–50). The required support can be medical, such as surgical repair, pain management, and physical therapy, as well as emotional or psychological, given the potential impact of such injuries on mental health and well-being (48–50). Hence, there is a need for a comprehensive care bundle that encompasses both prevention and post-tear support. This ensures a holistic approach, reflecting a multifaceted understanding of the health needs and preferences of pregnant women, including those from ethnic minority groups.

## 5 Implications for practice and research

The findings indicate several priorities for maternity and postnatal care. Women should receive balanced and individualised information about OASI during pregnancy, followed by clear explanations of the injury, treatment, recovery and available support after birth. Post-OASI care should address psychological and emotional well-being alongside physical recovery, with appropriate referral or access to mental health and pelvic health services where required. Greater consistency in communication and postnatal advice across healthcare professionals may also reduce uncertainty and distress.

Culturally responsive care is important when supporting women from diverse ethnic backgrounds. Healthcare professionals should recognise that cultural expectations and stigma may influence women's willingness to discuss childbirth complications, psychological distress or support needs, and should provide opportunities for sensitive and non-judgemental conversations.

Future research should include larger and more diverse samples across different maternity settings in England and the wider UK. Studies should seek to include women with different grades of OASI, including fourth-degree tears, as well as women who require interpreter support. Longitudinal research is also needed to explore how women's physical, psychological and social needs change over time. Future studies should evaluate interventions such as improved antenatal education, post-birth debriefing, culturally responsive psychological support and integrated post-OASI follow-up. The perspectives of partners and families should also be explored.

## 6 Transferability of the findings

As this was a qualitative study involving a small sample of ethnic minority women recruited in Leicester, the findings are not intended to be statistically generalisable to all women experiencing OASI. However, several issues identified, including inadequate preparation for OASI, difficulties in healthcare communication, psychological consequences of traumatic childbirth, cultural barriers to discussing mental health and inconsistencies in postnatal support, may be relevant to women receiving maternity care in other ethnically diverse NHS settings in England and the wider UK. The extent to which the findings are transferable will depend on similarities in population characteristics, maternity-service organisation and local cultural and healthcare contexts. Further research across different geographical and healthcare settings is therefore needed to determine whether these patterns are experienced more widely.

## 7 Study strength and limitation

This study has several limitations. The sample size was determined pragmatically by the number of eligible women who responded to the study invitation and agreed to participate during the recruitment period; no formal *a priori* sample-size calculation, information-power assessment or data-saturation criterion was used. Consequently, the small sample of ten women from ethnic minority backgrounds in Leicester may not fully capture the diversity of experiences among women affected by OASI. The geographical focus on Leicester also limits the transferability of the findings to women in other regions or healthcare settings.

Recruitment was conducted electronically, which may have limited participation among women with restricted digital access or technological skills. In addition, because the study information, consent process and FGD were conducted in English and interpreters were not used, women who were unable to participate sufficiently in English were excluded. This may have limited the representation of women who experience language-related barriers when accessing maternity services. Fathers or partners were also not included, and their perspectives on the impact of OASI and traumatic childbirth were therefore not captured.

Although the FGD encouraged interaction and the sharing of common experiences, the group format may have discouraged some women from discussing sensitive or intimate experiences that they might have been more comfortable disclosing during individual interviews. The clinician-led nature of the FGD may also have influenced participants’ willingness to openly discuss negative experiences of healthcare professionals or maternity services, and some accounts may therefore have differed from those that might have been elicited by an independent non-clinical researcher.

Women with either third- or fourth-degree perineal tears were invited to to participate, but all participants reported experiencing a third-degree tear. The study therefore does not capture the experiences of women with fourth-degree tears. Information on the specific classification of third-degree tears (3a, 3b or 3c) was not collected, which limits exploration of whether experiences differed according to the severity of OASI.

Furthermore, the educational presentation on ethnic disparities in third- and fourth-degree tears that preceded the FGD may have influenced participants’ responses by directing attention towards particular issues and potentially shaping the subsequent discussion. To enhance the trustworthiness of the findings, member checking was undertaken to ensure that the identified themes reflected participants’ perspectives.

## 8 Conclusion

This study highlights the urgent need to improve maternal care for women with OASI, particularly those from ethnic minority backgrounds. The findings highlight gaps in prenatal education, postpartum support, and healthcare communication**,** which contribute to both physical and psychological distress. Addressing these gaps requires a more comprehensive, patient-centered approach to OASI care. To improve maternal outcomes, healthcare systems should enhance prenatal education on OASI, implement culturally sensitive postpartum care, and standardize communication practices**.** Future research should focus on developing targeted interventions, integrating long-term physical and mental health support, and fostering inclusive maternity care policies**.** By prioritizing these efforts, healthcare providers can ensure more equitable and effective care for women recovering from OASI**.**

## Data Availability

The original contributions presented in the study are included in the article/Supplementary Material, further inquiries can be directed to the corresponding author.
